# Effect of Metal Oxide Nanoparticles on the Chemical Speciation of Heavy Metals and Micronutrient Bioavailability in Paddy Soil

**DOI:** 10.3390/ijerph17072482

**Published:** 2020-04-05

**Authors:** Wei Zhang, Jinghua Long, Jie Li, Meng Zhang, Xingyin Ye, Wenjing Chang, Hui Zeng

**Affiliations:** 1 School of Urban Planning and Design, Peking University Shenzhen Graduate School, Shenzhen 518055, China; zhangdaw007@heuet.edu.cn (W.Z.); zhangmeng@pkusz.edu.cn (M.Z.); yexingyin@pku.edu.cn (X.Y.); changwj@pkusz.edu.cn (W.C.); 2 School of Public Administration, Hebei University of Economics and Business, Shijiazhuang 050061, China; longjh_jm@heuet.edu.cn; 3 College of Land and Environment, Shenyang Agricultural University, Shenyang 110866, China; lijie@syau.edu.cn; 4 College of Urban and Environmental Sciences, Peking University, Beijing 100871, China

**Keywords:** metal oxide nanoparticles, heavy metal, bioavailability, paddy soil

## Abstract

The effects of engineered nanoparticles (ENPs) on heavy metal fate and biotoxicity in farmland soil are mostly unknown. A flooding–drying simulation experiment was conducted to study the effects of three typical metal oxide nanoparticles (TiO_2_-NPs, ZnO-NPs and CuO-NPs) on the chemical speciation of heavy metals and micronutrient bioavailability in paddy soil. The results showed that the addition of ZnO-NPs and CuO-NPs caused significant increases in soil pH, Eh and EC after a 90-d flooding–drying process. ZnO-NPs and CuO-NPs addition caused clearly increase in the Zn and Cu concentrations in the acid-soluble fraction, Fe/Mn oxides-bound fraction and organic-bound fraction, leading to higher bioavailability in the soil. DTPA-extractable Zn and Cu increased to 184.6 mg kg^−1^ and 145.3 mg kg^−1^ in the maximum ZnO-NPs and CuO-NPs concentration treatments (500 mg kg^−1^). TiO_2_-NPs promoted the transformation of Mn from a Fe/Mn oxides-bound fraction to an acid-soluble fraction. Soil Cd bioavailability obviously decreased in the TiO_2_-NPs treatment but increased in the ZnO-NPs and CuO-NPs treatments.

## 1. Introduction

With the wide use of nanomaterials, large numbers of engineered nanoparticles (ENPs) are discharged into the water and soil system [1,2]. TiO_2_-NPs, ZnO-NPs and CuO-NPs are common metal oxide nanoparticles (MNPs) used in plant growth, cosmetics, surface coating and sunscreens, etc., and the biotoxicity and the environmental risk of these metal oxide nanoparticles has caused extensive concern in recent years [3,4]. For example, CuO nanoparticles caused a significant decrease in rice seed germination percentage [5], and 1000 mg L-1 ZnO-NPs could reduce the root length of corn and cucumber [6]. The biotoxicity of nanoparticles depends on its particle size, surface charge and concentration [7]. 

Due to the special surface properties of nanoparticles, they can influence the migration and bioavailability of co-existing contaminants in the water and soil environment. [2,8,9,10]. For example, Shrestha et al. (2015) found that multiwalled carbon nanotubes (MWCNTs) significantly reduced the toxicity and bioavailability of polycyclic aromatic hydrocarbons (PAHs) in the soil microbial community, and the effects were dependent on the soil organic matter content [11]. Zero-valent iron nanoparticles (nZVI), often used in water and soil pollution remediation, could effectively reduce contaminants’ bioavailability [12,13]. Graphene oxide (GO) could promote the transformation of arsenic (As) from As(V) to high-toxicity As(III) in soil, and significantly influence the morphological and biochemical characteristics of rice [14]. TiO_2_-NPs significantly reduced the toxic effects of tetracycline on rice in terms of its fresh biomass and levels of antioxidant enzymes [15]. Most of the studies about the effect of nanoparticles on heavy metals behaviour were conducted in hydroponic conditions, and only a few were conducted in soil conditions [4,16,17,18]. 

MNPs would dissolve and release metal ions after entering the aquatic system or under soil environment. For example, Franklin et al. demonstrated that ZnO-NPs rapidly dissolved in a freshwater medium (pH 7.6) and a buffer solution (0.01 M Ca(NO_3_)_2_, pH 7.5), and the concentrations of Zn^2+^ reached 6 mg·L^−1^ and 16 mg·L^−1^ in 6 h and 72 h, respectively [19]. Some studies concluded that the toxicity of CuO-NPs depends on its solubility in soil [20,21]. Addition of 500 mg kg CuO-NPs caused the labile Cu in the soil to increase by 206 mg kg after a 42 day pot experiment [22]. At present, the connection between MNP dissolution, the resulting dose of metal ions, and changes in the chemical speciation of heavy metals and its influence on soil micronutrient bioavailability have not been well elucidated due to a lack of appropriate characterization of the dissolution of the NPs in soil. Moreover, due to the complexity of soil media, it is hard to explain the mechanism of various phenomena in soil experiments.

The current study focused on the environmental behaviour of MNPs in paddy soil. A flooding–drying simulation experiment was conducted to study the effect of three typical metal oxide nanoparticles (TiO_2_-NPs, ZnO-NPs and CuO-NPs) on the chemical speciation of heavy metals and the micronutrient bioavailability in paddy soil. The results will further our understanding on the nanoparticles behaviour in soil and provide a scientific assessment for the agricultural ecological risk of engineered nanoparticles.

## 2. Materials and Methods 

### 2.1. Soil Characteristics

The paddy soil used in this research was sampled from Huizhou, Guangdong Province, China. Soil samples were air dried and sieved to less than 2 mm. The contents of organic matter (OM) and cation exchange capacity (CEC) were 24.5 g·kg^−1^, and 8.50 cmol·kg^−1^, respectively. The concentrations of Fe, Mn, Cu, Zn and Cd in soil were 9.43 g·kg^−1^, 92.7 mg·kg^−1^, 22.6 mg·kg^−1^, 56.7 mg·kg^−1^ and 2.70 mg·kg^−1^, respectively (supporting information (S1) Table S1).

### 2.2. Nanoparticles Characteristics

The metal oxide nanoparticles (TiO_2_-NPs, ZnO-NPs and CuO-NPs) used in this research were purchased from Nanjing XFNANO Materials Tech Co., Ltd, (Nanjing, China), and the purity of the nanomaterials is above 99.9%. The characterizations of nanoparticles were performed in the Key laboratory of nano-micro materials, Peking University Shenzhen Graduate School. The size of the metal oxidize nanoparticles was detected by a field emission scanning electron microscope (ZEISS SUPRA^®^ 55, Carl Zeiss, Jena, Germany), and the specific surface area was detected by an Accelerated Surface Area and Porosimetry System (ASAP 2020 HD88, Micromeritics, Atlanta, GA, USA). The diameter size of these MNPs in the range of 20–40 nm. The specific surface areas of the TiO_2_-NPs, ZnO-NPs and CuO-NPs were 77.4 m^2^·g^−1^, 21.5 m^2^·g^−1^ and 131 m^2^·g^−1^, respectively.

### 2.3. Experimental Design

Three different concentrations (50, 100 and 500 mg kg) of MNPs (TiO_2_-NPs, ZnO-NPs and CuO-NPs) were uniformly added into the soil (TiO_2_-NPs: T50, T100, T500; ZnO-NPs: Z50, Z100, Z500; CuO-NPs: C50, C100, C500). The treatment with no nanoparticles added was set as the control (CK). Each treatment was conducted in triplicate.

Metal oxide nanoparticles powder (0.05 g, 0.10 g and 0.50 g) was finely dispersed with air-dried soil (9.95 g, 9.90 g and 9.50 g) in a ceramic plate, stir evenly. Next, the mixture was placed into a plastic bottle container (2.5 L) with 90 g air-dried soil sample, mixed thoroughly with a rotary agitator (YKZ-08) for 10 min (25 r/min). Then, 900 g air-dried soil sample was added into the container, and shaken for 30 min. 

The 250 g mixed soil sample was placed into a bottle container (10 cm diameter × 22 cm height), and 200 mL deionized water was added to the container. Soils were incubated for 90 d at 25 °C in an incubator (SHP-250JB, Changzhou Putian instrument manufacturing Co., Ltd, Changzhou, China). The flooding–drying experiment simulated rice growth conditions, the start time was considered the seeding plant time, and the 30th, 60th and 90th d were considered the tillering stage, booting stage and heading stage, respectively. In the first 60-day period, the water layer depth was 3 cm above the soil surface, and we drew a line on the bottle surface; deionized water was added every day to achieve the flooding condition and keep the water depth stable. At the last 30-day period, the soil was treated in the drying-wetting condition alternately, and deionized water was supplemented every ten days [23]. After the 90-d experiment, the soil samples were collected, air dried and sieved to less than 2 mm.

### 2.4. Analytical Methods

Soil pH, electrical conductivity (EC) and soil redox potential (Eh) were measured by an ion analyser (Thermo-Orion, Beverly, MA, USA) with a combination electrode (pH electrode, conductivity meter and oxidation reduction potential electrode) (9678BNWP, Thermo-Orion, Beverly, MA, USA) combined with a Super Ross vitreous reference electrode (800500U, Thermo-Orion, Beverly, MA, USA).

Soil samples were digested by a HCl-HNO_3_-HF-HClO_4_ mixed-acid digestion method. 0.5 g air-dried soil samples were digested with 35 ml mixture of hydrochloric acid (HCl), nitric acid (HNO_3_), hydrofluoric acid (HF) and perchloric acid (HClO_4_) (5 mL, 15mL, 10 mL, 5mL) [2]. All the acid used in the digestion process was in a guaranteed reagent level (GR, 99.8%), and the concentrations of the acids used in this digestion were 36%–38%, 65%–68%, ≥40% and 70.0%–72.0% for hydrochloric acid (HCl), nitric acid (HNO_3_), hydrofluoric acid (HF) and perchloric acid (HClO_4_), respectively.

Improved BCR sequential extraction method was used to analyse the chemical speciation of heavy metals in soil, and the details of this method were described in Nemati et al. [24].

CaCl_2_ and diethylenetriaminepentaacetic acid solution (DTPA) extraction are two common methods used to predict the bioavailability of elements in soil. CaCl_2_-extractable is considered “readily available” to plants, and DTPA-extractable is considered “potentially available” fraction. In this study, the bioavailability of the micronutrients in the soil was extracted with calcium chloride solution (CaCl_2_, 0.01 M) and (DTPA) [25]. Solutions was filtered with a Millipore filter (0.45 μm), and the concentrations of the elements in the solution were determined by inductively coupled plasma mass spectrometry (Agilent 7700x ICP-MS, Agilent Technologies, Santa Clara, CA, USA). All samples were measured in triplicate, and the analytical error for selected elements was smaller than 3%.

### 2.5. Data Analysis

Results were presented as mean ± standard deviation (SD) of three independent experiments. All the experimental data was analysed by SPSS software (SPSS Statistics 20, IBM, Armonk, NY, USA), one-way ANOVA followed by the Tukey-HSD test was used to analyse the differences among various groups. *p* < 0.05 indicated a significant difference. 

## 3. Results and Discussion

### 3.1. Effect of MNPs Addition on Soil Properties

The factors that influenced the heavy metal chemical speciation in the soil included pH, redox potential (Eh), organic matter, cation exchange capacity (CEC), iron manganese oxides and microorganisms. The nanoparticles affected the physical and chemical properties, the components, and the microbial function and community structure of the soil [26,27,28], and they subsequently influenced the heavy metal transformation and bioavailability of the soil. 

**Figure 1:**
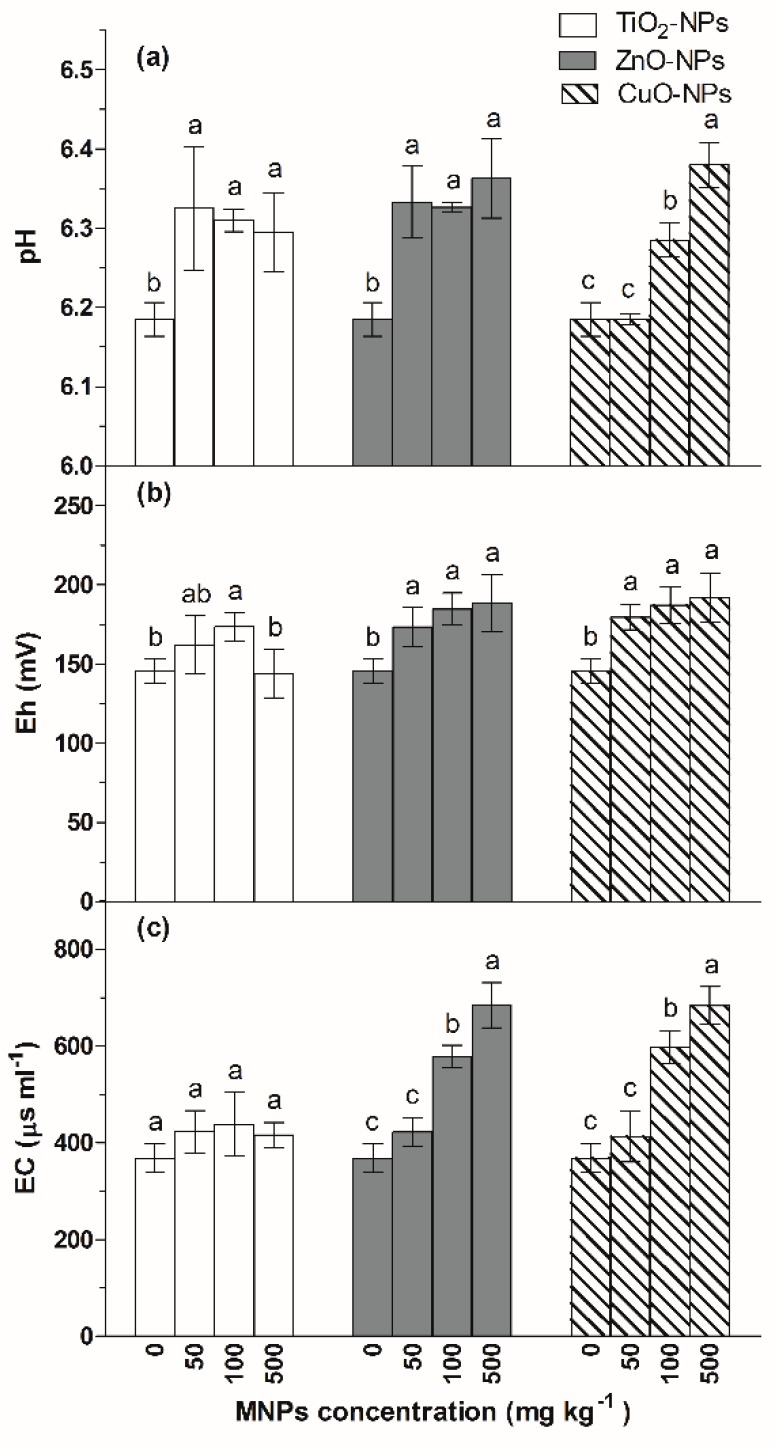
Changes in the values of soil pH (**a**), redox potential (Eh) (**b**) and electric conductivity (EC) (**c**) of the soil exposed to different MNPs. Different letters above each column indicate significant difference (*p* < 0.05) between various treatments in same group.

As shown in Figure 1a, the addition of ZnO-NPs and CuO-NPs caused significant (*p* < 0.05) increases in the values of soil pH after the 90-d flooding–drying process. Shi et al. (2018) found that CuO nanoparticles could significantly increase the soil pH values, which was consistent with our results [25]. Take CuO NPs as an example, after entering the soil, the following chemical reactions will occur:

$$CuO+2H^{+}\overset{\;}{↔}Cu^{2+}+H_{2}O$$

$$CuO+2H^{+}\overset{\;}{↔}Cu{\left(HO\right)}^{+}$$

CuO could consume H^+^ in soil solution, generate Cu^2+^ and Cu(HO)^+^, and consequently caused the increase in soil pH values. ZnO NPs has a similar reaction process in soil. Cullen et al. (2011) also observed that metal nanoparticles significantly increased the soil pH [29].

Soil redox potential is an important indicator in paddy soil. The changes of soil Eh depend on the properties and concentrations of oxidized and reductive substances in soil. During the flooding period, soil microorganisms consume the oxygen in the soil, and the air oxygen can not diffuse into the soil and generate reductive substances, thereby, causing the decrease of soil Eh. As shown in Figure 1b, the addition of ZnO-NPs and CuO-NPs caused a slight increase in soil Eh. For instance, the soil Eh increased by 20~30 mV in the ZnO-NPs and CuO-NPs treated soil compared to that of the control. That was contrasted with Cullen et al.’s results, which demonstrated that nanoscale zerovalent iron decreased soil Eh. Frenk et al. (2013) found that a 1% CuO-NPs addition could decrease the soil Eh, while, after a biocidal treatment, the addition of CuO-NPs could significantly increase the soil Eh. Their results demonstrated that CuO-NPs could influence soil Eh by both chemical and biological actions [30]. In the flooding condition, the influence of chemical action may be more obvious than biological action, and CuO-NPs and ZnO-NPs could consume reductive substance (H^+^), causing the increase of soil Eh. 

Soil electric conductivity (EC) was determined by the soil soluble salt content and affected plant nutrient uptake and growth. As shown in Figure 1c, higher concentrations of ZnO-NPs and CuO-NPs (500 and 100 mg·kg^−1^ significantly (*p* < 0.05) increased soil EC values after the 90-d flooding–drying process. The soil EC value increased from 368 μs·mL^−1^ to 684 and 683.5 μs·mL^−1^ in high concentrations (500 mg·kg^−1^) of ZnO-NPs and CuO-NPs treatment, respectively. The dissolution of CuO-NPs and ZnO-NPs increased the Zn^2+^ and Cu^2+^ concentrations in the soil, also leading to the increase in soil EC. Previous studies have found that the addition of nanoscale CuO-NPs could significantly increase soil EC [31], partially supporting our observations. Due to the lower solubility of TiO_2_-NPs, the amount of available Ti in the soil was not detected, and the soil EC for the TiO_2_-NPs treatment did not evidently change compared to those of the ZnO-NPs and CuO-NPs. 

### 3.2. Effect of MNPs Addition on Heavy Metal Chemical Speciation

The agglomeration of MNPs in soil medium caused inhomogeneous distribution of MNPs. The total content of Cu in the soils with CuO-NPs’ addition and the Zn content in the soils with ZnO-NPs’ addition were shown in Table S2. The recovery of Cu for CuO-NPs addition soils in the range of 76%–81%, and the recovery of Zn in the range of 80%–86%.

**Figure 2:**
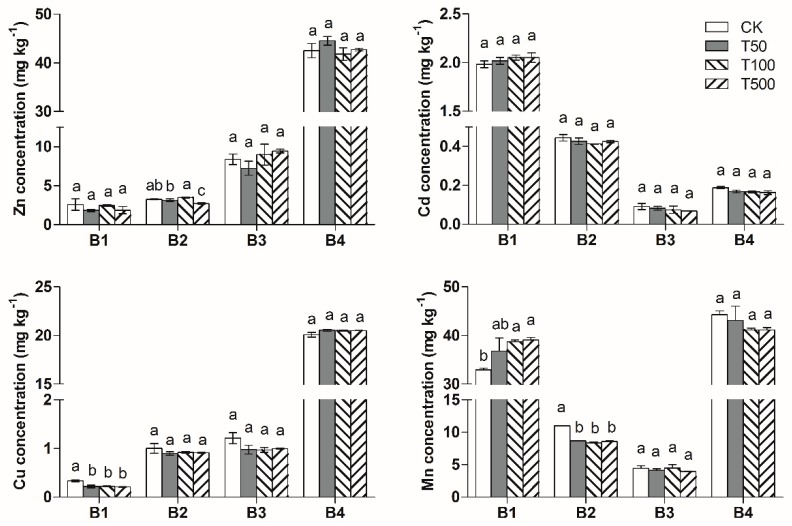
Changes in the chemical speciation of soil heavy metals with TiO_2_-NPs addition. B1: acid-soluble fraction; B2: Fe/Mn oxides-bound fraction; B3: organic-bound fraction; B4: residual fraction. Different letters above each column indicate significant difference (*p* < 0.05) between various treatments.

After the 90-d flooding–drying process, Cd in the control soil was mostly in the form of the acid-soluble fraction, accounting for 75.4% of the total concentration; the Fe/Mn oxide-bound fraction accounted for 16.6% of the total; the organic-bound fraction and residual accounted for 3% and 5%, respectively (Figure 2). Zn and Cu in the control treatment were mostly in the form of residuals, accounting for 70.0% and 88.7% of the total concentration, respectively, and the acid-soluble fraction accounted for only 4.5% and 1.5%, respectively. Mn in the control treatment mainly consists of the acid-soluble fraction and residual, accounting for 35.5% and 47.8% of total concentration, respectively. 

#### 3.2.1. TiO_2_-NPs

As shown in Figure 2, the organic bound fraction and residual of the Cu, Zn and Cd concentrations indicated no obvious effect from the TiO_2_-NPs treatment. The addition of TiO_2_-NPs significantly reduced the acid-soluble Cu; however, there was no significant difference between different concentration treatments.

For the TiO_2_-NPs treatment, the acid-soluble Mn in the soil increased with the increase in TiO_2_-NPs concentration. The acid-soluble fraction of Mn in soil was much higher in T500 and T100 treatment than that in the control. The Fe/Mn oxides-bound fraction of Mn was obviously lower compared to that of the control. Results indicated that the addition of TiO_2_-NPs promoted the transformation of Mn from a Fe/Mn oxides-bound fraction to an acid-soluble fraction. The possible mechanisms may be related with electrons (electron-hole pairs), which generated on the surface of TiO_2_-NPs, and the free electrons could promote the transformation of the Fe^3+^, Mn^4+^ and Mn^3+^ to Fe^2+^ and Mn^2+^. The released Fe^2+^ and Mn^2+^ were adsorbed on the surface of soil colloid and transformed into an acid-soluble fraction.

#### 3.2.2. ZnO-NPs

**Figure 3:**
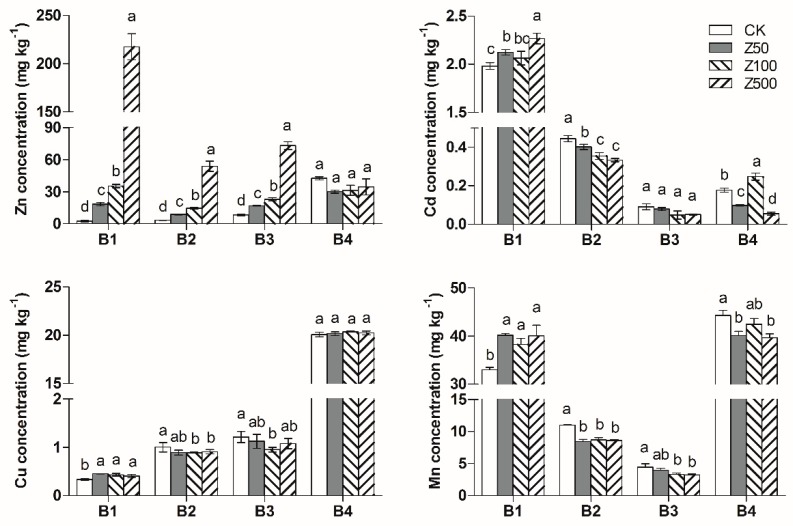
Changes in soil heavy metals chemical speciation with ZnO-NPs addition. B1: acid-soluble fraction; B2: Fe/Mn oxides-bound fraction; B3: organic-bound fraction; B4: residual fraction. Different letters above each column indicate significant difference (*p* < 0.05) between various treatments.

For the ZnO-NPs treatment, significant increases in the acid-soluble fraction, Fe/Mn oxides-bound fraction and organic-bound fraction of the Zn concentration were observed (Figure 3). Different concentrations of ZnO-NPs addition had no impact on the residual fraction of Zn in the soil. The acid-soluble fraction of Zn increased by 15.8, 32.7 and 215 mg·kg^−1^ compared to the control treatment for Z50, Z100 and Z500, respectively. Compared with the control, the Fe/Mn oxides-bound fraction of Zn in the Z50, Z100 and Z500 treatments increased by 5.54, 11.3 and 50.4 mg·kg^−1^, respectively, and the organic-bound fraction of Zn increased by 8.56, 14.6 and 64.7 mg·kg^−1^, respectively.

This outcome revealed that ZnO-NPs could dissolve in the soil and release ionic Zn, and approximately 40%~50% of the Zn ions existed in the acid-soluble fraction after the 90-d experiment, 12%~14% of the Zn ions existed in the Fe/Mn oxides-bound fraction, and 14%~21% of the Zn ions existed in the organic-bound fraction. 

For the ZnO-NPs treatment, the acid-soluble Cd increased in response to the increase in the nanoparticle concentration. The acid-soluble fraction of Cd in the soil was much higher in the Z500 treatment than that in the Z50 and Z100 treatments. The Fe/Mn oxides-bound fraction of Cd in the ZnO-NPs treatment was statistically (*p* < 0.05) significantly lower than that in the control treatment.

The addition of ZnO-NPs clearly increased the acid-soluble Mn concentrations and decreased the Fe/Mn oxides-bound fraction and organic fraction of the Mn concentrations. Normally, the iron and manganese oxides would dissolve in reductive conditions and undergo oxidative precipitation in oxidation conditions. In this research, the iron and manganese oxides in the soil were deoxidized into Fe^2+^ and Mn^2+^ under flooding conditions, and iron Mn was released into the surface water. During the drying process, the Fe^2+^ and Mn^2+^ would gradually oxidize and precipitate. Due to the oxidation time being relatively short in our experiment, it caused the Fe/Mn oxides-bound fraction of Mn in the soil to decrease, and the acid-soluble Mn to increase.

#### 3.2.3. CuO-NPs

**Figure 4:**
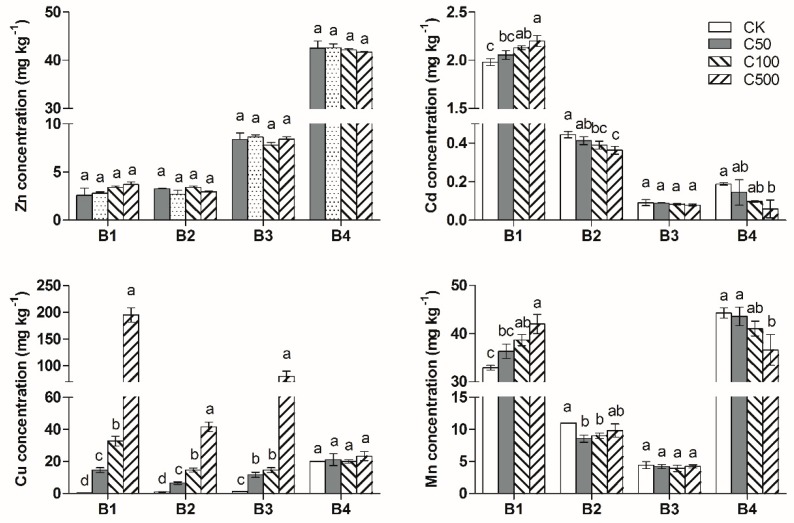
Changes in soil heavy metals chemical speciation with CuO-NPs addition. B1: acid-soluble fraction; B2: Fe/Mn oxides-bound fraction; B3: organic-bound fraction; B4: residual fraction. Different letters above each column indicate significant difference (*p* < 0.05) between various treatments.

As shown in Figure 4, the addition of CuO-NPs had no significant effect on changes in Zn chemical speciation. The addition of CuO-NPs obviously increased the acid-soluble Cd and Mn concentrations and decreased the Fe/Mn oxides-bound fraction of the Cd and Mn concentrations. The influence became more obvious as the CuO-NPs concentration increased.

For the CuO-NPs treatment, significant increases in the acid-soluble fraction, Fe/Mn oxides-bound fraction and organic-bound fraction of the Cu concentration were observed. The acid-soluble fraction of Cu increased by 14.7, 32.7 and 195 mg·kg^−1^ compared to that of the control treatment for C50, C100 and C500, respectively. This outcome revealed that the CuO-NPs could dissolve in the soil and release ionic Cu; after the 90-d experiment, approximately 35%~48% of the Cu ions existed as the acid-soluble fraction, 10%~18% of the Cu ions existed as the Fe/Mn oxides-bound fraction, and 20%~29% of the Cu ions existed as the organic-bound fraction. For changes in Cu chemical speciation in the soil, the proportion of the acid-soluble fraction increased, while the proportion of Fe/Mn oxides-bound fraction and organic-bound fraction decreased, in response to the increase in the CuO-NPs concentration.

Soil redox potential was one of the key factors influencing the heavy metal chemical speciation, the acid-soluble heavy metal concentrations in soil would increase under oxidation condition. In this research, the addition of CuO-NPs and ZnO-NPs increased the soil Eh, and then promoted the transformation of Cd and Mn from a Fe/Mn oxides-bound fraction to an acid-soluble fraction. In addition, the adsorption of soil microorganisms on heavy metals was also an important factor influencing the heavy metal chemical speciation. Soil microorganisms could adsorb heavy metals and affect the mobility of heavy metals in the soil environment. Many studies have shown that the addition of nanoparticles in soil could affect soil microbial biomass and soil enzyme activities [23]. MNPs could influence the heavy metal chemical speciation by affecting the soil microorganisms. In this research, the changes of Cd and Mn chemical speciation may also be related with the change of soil microorganisms which is caused by the addition of CuO-NPs and ZnO-NPs.

After the 90-d flooding–drying process, more than half of the ZnO-NPs and CuO-NPs dissolved and released ionic Zn and Cu into the soil. The dissolution of CuO-NPs and ZnO-NPs clearly increased the Cu and Zn concentrations in the acid-soluble fraction, Fe/Mn oxides-bound fraction and organic fraction but have no significant impact on the residual fraction. Total concentrations of Zn and Cu reached approximately 400 mg·kg^−1^ in the maximum nanoparticle concentration treatments (500 mg·kg^−1^). Although the Zn/Cu concentration did not exceed the threshold of the Chinese environmental quality standard for soils (GB15618-1995) (500 mg·kg^−1^), there was a potential environmental risk as the MNPs’ concentrations increased. 

### 3.3. Effect of MNPs Addition on Soil Cd and Micronutrient Bioavailability

**Figure 5:**
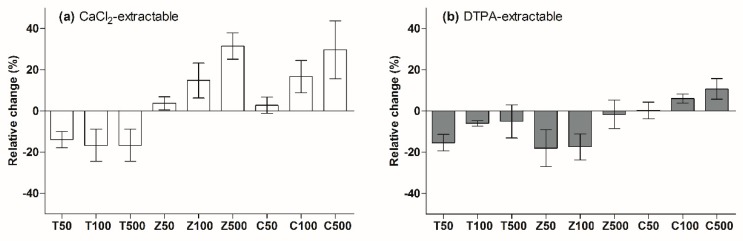
Relative change in soil Cd bioavailability after the addition of nanoparticles.

Two standard extraction methods (CaCl_2_ and DTPA) were used to assess the change in soil Cd and micronutrient (Fe, Mn, Cu, Zn) bioavailability after the addition of MNPs. As shown in Figure 5, the addition of TiO_2_-NPs clearly decreased the soil Cd bioavailability and the Cd concentration extracted by CaCl_2_ and DTPA, which decreased by 13.8%~16.8% and 5.0%~15.4%, respectively, compared to that of the control treatment. 

For the ZnO-NPs treatment, the CaCl_2_-extractable Cd concentration increased by 3.7%~31.5%, and the increase was proportional to the ZnO-NPs concentration. However, ZnO-NPs significantly decreased the DTPA-extractable Cd concentration. The addition of CuO-NPs caused significant increases in the soil Cd bioavailability, and the increase was in proportion to the CuO-NPs concentration. Compared with the control treatment, the CaCl_2_-extractable Cd concentration increased by 2.8%~29.6%, and the DTPA-extractable Cd concentration increased by 0.3%~10.7%.

The bioavailability of Cd in the soils increased in response to the increases in the ZnO-NPs/CuO-NPs concentrations because the dissolution of the ZnO-NPs/CuO-NPs released Zn/Cu ions, and the competitive adsorption between Zn/Cu and Cd reduced the adsorption capacity of Cd in the soil [32]. The CaCl_2_-extractable Cd had a significant correlation (*p* < 0.05) with acid-soluble Cd content in soil, which indicated that CaCl_2_ extraction was more suitable for the evaluation of the soil Cd bioavailability in this study.

**Figure 6:**
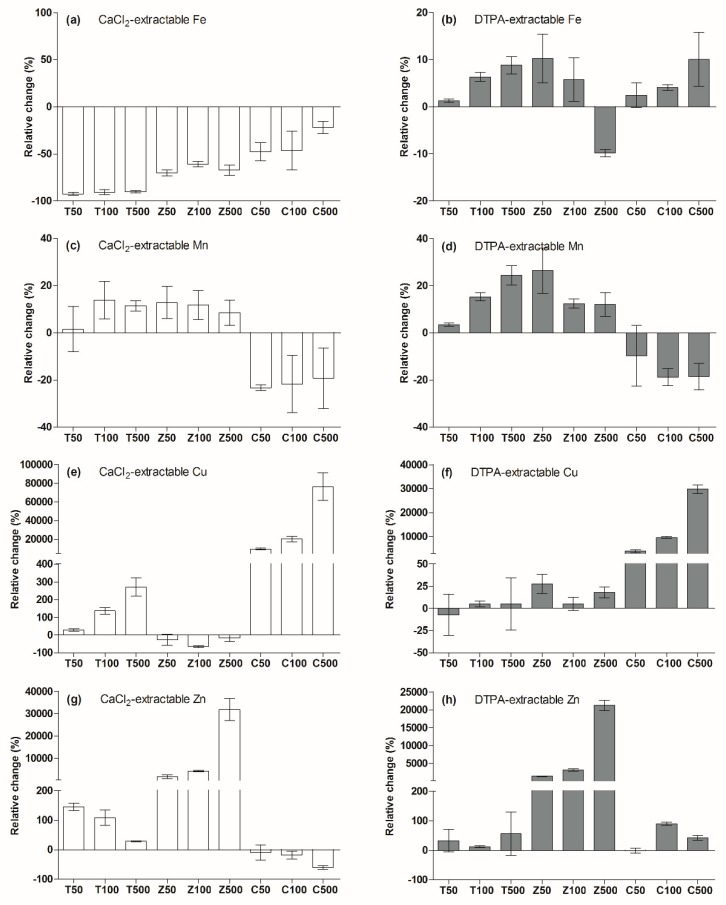
Relative change in soil micronutrient bioavailability after the addition of nanoparticles.

Iron, manganese, copper and zinc are essential crop micronutrients. A deficiency may lead to reduced disease resistance and decreased crop yields [24,33]. However, at high concentrations, these elements can also be toxic to plants and the surrounding microbial communities [22,34,35]. As shown in Figure 6a,b, the influence of MNPs on CaCl_2_-extractable Fe and DTPA-extractable Fe was totally different. The MNPs addition caused a decrease of the CaCl_2_-extractable Fe concentration, but an increase of the DTPA-extractable Fe concentration.

Both the TiO_2_-NPs and ZnO-NPs addition caused significant increases in Mn bioavailability. As shown in Figure 6c,d, in the T100 and T500 treatments, CaCl_2_-extractable Mn increased by 13.8% and 11.4%, respectively, and DTPA-extractable Mn increased by 15.3% and 24.4%, respectively. ZnO-NPs addition caused CaCl_2_-extractable Mn increased by 8.5%~12.8%, and DTPA-extractable Mn increased by 12.0%~26.3%. However, the addition of CuO-NPs reduced the soil Mn bioavailability compared with that of the control treatment, CaCl_2_-extractable Mn decreased by 19.3%~23.3%, and DTPA-extractable Mn increased by 9.7%~18.8%.

For copper, with the increase in the TiO_2_-NPs concentration, the soil CaCl_2_-extractable Cu increased by 30%~270% (Figure 6e). With the addition of ZnO-NPs, the CaCl_2_-extractable Cu decreased by 17.0%~64.9%, and DTPA-extractable Cu increased by 5.2%~27.9% (Figure 6f). CuO-NPs dissolved and released ionic Cu after addition to the soil. In the C50, C100 and C500 treatments, CaCl_2_-extractable Cu increased from 0.003 mg·kg^−1^ to 0.32, 0.68 and 2.6 mg·kg^−1^, respectively, and DTPA-extractable Cu increased from 0.05 mg·kg^−1^ to 19.0, 47.0 and 145.3 mg·kg^−1^, respectively.

TiO_2_-NPs addition obviously increased the soil Zn bioavailability, CaCl_2_-extractable Zn increased by 28.6%~145.5%, and DTPA-extractable Zn increased by 12.2%~56.1% (Figure 6g,h). ZnO-NPs dissolved and released ionic Zn after the addition to the soil, and the increase in the Zn concentration was in proportion to the ZnO-NPs concentration. In the Z50, Z100 and Z500 treatments, CaCl_2_-extractable Zn increased from 0.055 mg·kg^−1^ to 0.98, 2.4 and 17.6 mg·kg^−1^, respectively, and DTPA-extractable Cu increased from 0.9 mg·kg^−1^ to 12.8, 28.4 and 184.6 mg·kg^−1^, respectively. In the C100 and C500 treatments, CaCl_2_-extractable Zn decreased by 18.2% and 60.0%, respectively, but in contrast, DTPA-extractable Zn increased by 90.2% and 42.2%, respectively.

The effect of the MNPs on the heavy metal bioavailability of the soil depended on the soil properties, the dissolving capacity and diameter of the nanoparticles, the test conditions, etc. Adsorption studies have shown that MNPs affect the fate and transport of contaminants in aqueous conditions [36,37,38]. In this study, the results showed that these three MNPs exhibited different influences on the chemical speciation of heavy metals and micronutrient bioavailability in the soil. TiO_2_-NPs could affect the heavy metals and micronutrients bioavailability attributed to the special surface properties of nanoparticle, however, the effect of ZnO-NPs/CuO-NPs was due to the Zn^2+^ and Cu^2+^ that released from the dissolution of the ZnO-NPs/CuO-NPs.

In soil conditions, nanoparticles may aggregate in the surface of the soil and have no significant impact on the surface area of soil [37]. On the other hand, heavy metals adsorbed in colloids could be transported with the colloids in soils, leading to higher mobility and bioavailability [37,38], and the nanoparticles exhibited similar behaviour and effects in the environment compared to the colloids [39]. Thus, it is necessary to conduct further research on the environmental behaviour of nanoparticles in soil system.

## 4. Conclusions

In this study, the chemical speciation and bioavailability of heavy metals and micronutrients were observed. ZnO-NPs and CuO-NPs caused significant increases in the values of soil pH and EC after the 90-d flooding–drying process. ZnO-NPs and CuO-NPs addition caused clear increases in the Zn and Cu concentrations in the acid-soluble fraction, Fe/Mn oxides-bound fraction and organic-bound fraction, leading to higher bioavailability in the soil. DTPA-extractable Zn and Cu increased to 184.6 mg·kg^−1^ and 145.3 mg·kg^−1^, respectively, in the maximum ZnO-NPs and CuO-NPs concentration treatments (500 mg·kg^−1^). These three MNPs exhibited different influences on soil micronutrient bioavailability. Soil Cd bioavailability was obviously decreased in the TiO_2_-NPs treatment but increased in the ZnO-NPs and CuO-NPs treatments. The addition of TiO_2_-NPs and ZnO-NPs obviously increased soil Mn bioavailability, while the addition of CuO-NPs reduced soil Mn bioavailability. Further efforts are needed to investigate the environmental behaviour of nanoparticles in soil systems.

## Supplementary Materials

The following are available online at https://www.mdpi.com/1660-4601/17/7/2482/s1, Table S1: Basic physicochemical properties of paddy soils, Table S2: The recovery of Cu and Zn in the soils with CuO-NPs and ZnO-NPs addition.
