# Supplementary material for: Effect of Metal Oxide Nanoparticles on the Chemical Speciation of Heavy Metals and Micronutrient Bioavailability in Paddy Soil
Source: Int J Environ Res Public Health. 2020 Apr 5;17(7):2482. doi: 10.3390/ijerph17072482 (PMC7177736; doi:10.3390/ijerph17072482)
Supplement: Supplementary file 1 [file ijerph-17-02482-s001.pdf]

Supplementary data

Effect of metal oxide nanoparticles on the chemical speciation of heavy metals and micronutrient bioavailability in paddy soil

Table S1. Basic physicochemical properties of paddy soils

Test Items	Paddy Soil
pH	6.20±0.10
Organic content (%)	2.45±0.09
Sand fraction (%)	34.0±0.9
Silt fraction (%)	45.5±1.5
Clay fraction (%)	20.5±1.9
Cation exchange capacity (cmol/kg)	8.50±0.07
Total N (g/kg)	2.25±0.70
Total P (g/kg)	0.46±0.09
Total K(g/kg)	12.35±3.66
Total Fe (g/kg)	9.43±0.72
Total Mn (mg/kg)	92.69±8.14
Total Cu (mg/kg)	22.62±3.17
Total Zn (mg/kg)	56.74±3.19
Total Cd (mg/kg)	2.70±0.24

Table S2. The recovery of Cu and Zn in the soils with CuO-NPs and ZnO-NPs addition

Treatment	ZnO-NPs			CuO-NPs		
	Z50	Z100	Z500	C50	C100	C500
Measured value (mg kg^{-1})	74 ± 1.4	104 ± 6.3	370 ± 17	54 ± 4.1	82 ± 7.7	340 ± 21
Theoretical value (mg kg^{-1})	97	137	457	63	103	423
Recovery (%)	76 ± 1.4	76 ± 4.6	81 ± 3.7	86 ± 6.5	80 ± 7.5	80 ± 5.0
